# Allele specific expression in Alzheimer's disease

**DOI:** 10.1002/alz.71558

**Published:** 2026-06-11

**Authors:** Zishan Wang, Delowar Hossain, Judy Jiaru Wang, Varun R. Subramaniam, Bin Zhang, Minghui Wang, Kuan‐lin Huang

**Affiliations:** ^1^ Department of Genetics and Genomic Sciences, Center for Transformative Disease Modeling, Tisch Cancer Institute, Icahn Institute for Data Science and Genomic Technology Icahn School of Medicine at Mount Sinai New York New York USA; ^2^ Division of Experimental Medicine McGill University Montréal Québec Canada; ^3^ College of Arts and Sciences Cornell University Ithaca New York USA

**Keywords:** allele specific expression, Alzheimer's disease, multi‐omics

## Abstract

**INTRODUCTION:**

Allele‐specific expression (ASE), preferential expression of one allele at a heterozygous locus, is implicated in various brain diseases but remains largely uncharacterized in Alzheimer's disease (AD).

**METHODS:**

We performed a genome‐wide characterization of ASE variants across seven brain regions of 2,231 AD and Control patients from Mount Sinai Brain Bank (MSBB) and Religious Orders Study/Memory and Aging Project (ROSMAP) cohorts and investigated cell‐type–specific activity via single‐cell analysis.

**RESULTS:**

We identified 56,136 unique ASE variants that were enriched in imprinted chromosomal regions, e.g., chr6, chr14q32, and chr15q11. ASE variants were also found in exons of known AD‐associated genes, including apolipoprotein E *(APOE)*, *CLU*, *CTSB*, and *HLA‐DRB1*. Forty variants exhibited AD‐associated ASE, and the affected genes, including *SLC12A5, SYT13, and TOMM7*, were predominantly downregulated in multiple cell types, including astrocytes, excitatory neurons, and oligodendrocytes.

**DISCUSSION:**

We provided a detailed landscape of ASE in AD, uncovering novel functional variants and highlighting their potential cell‐type–specific contributions to disease pathogenesis.

## Highlights

- 56,136 allele‐specific expression (ASE) variants identified across seven brain regions in 2,231 tissue samples
- ASE variants enriched in imprinted regions on chr6, chr14q32, and chr15q11
- Forty Alzheimer's disease (AD)‐associated ASE variants discovered across two independent cohorts
- Affected genes predominantly downregulated in multiple brain cell types
- ASE bridges genome‐wide association studies (GWAS) loci in apolipoprotein E (APOE), CLU, and HLA‐DRB1 to expression changes

## 1 BACKGROUND

Alzheimer's disease (AD), the leading cause of dementia, accounts for 50%–70% of dementia cases.^1^ Neurological conditions, including AD and other dementias, are the foremost contributors to disability‐adjusted life years (DALYs).^2^ Most AD cases are late‐onset Alzheimer's disease (LOAD) occurring after age 65, a complex pathology with an estimated heritability of up to 80%.^3^ Although several anti‐amyloid beta (Aβ) monoclonal antibodies have been approved since 2021 by the United States Food and Drug Administration (FDA) to treat AD,^4^ existing therapies provide only partial symptom relief without halting disease onset or progression. As the global population aged 65 or older expands at an unprecedented speed,^5^ there is an urgent need to decipher the genetic underpinnings of LOAD and identify biomarkers or targets for effective therapy.

Genome‐wide association studies (GWAS), together with transcriptomic and proteomic analyses, have identified numerous risk loci, dysregulated genes, and perturbed networks in AD.^3^, ^6^, ^7^, ^8^, ^9^ However, a significant gap remains in our understanding of the causal genetic variants that drive functional expression effects and their associated signaling pathways. This knowledge gap presents a substantial barrier to translating genetic associations into actionable therapeutic interventions. Allele‐specific expression (ASE), where one allele at a heterozygous locus is preferentially expressed at the RNA level, offers a powerful approach for interrogating these mechanisms. ASE has been implicated in various genes,^10^ frequently affecting established imprinted regions. ASE quantifies expression variation between the two gene copies at a heterozygous site,^11^ enabling the identification of *cis*‐regulatory elements influencing gene expression. ASE has proven instrumental in elucidating functional genes in other brain disorders, including Parkinson's disease^12^ and autism spectrum disorder (ASD).^13^ In AD, ASE has been leveraged to identify ASE quantitative trait loci for 33 known AD‐associated variants; however, this analysis was not performed on a whole‐genome scale and thus unable to systematically identify novel genetic loci.^14^ These findings underscore the potential of a systematic characterization of ASE on a genome‐wide level to unravel mechanisms underlying AD pathogenesis.

Here, we systematically identified and characterized ASE variants by analyzing multi‐omics data across seven brain regions in AD patients, with the majority being aged 65 or older, from the Mount Sinai Brain Bank (MSBB) and the Religious Orders Study/Memory and Aging Project (ROSMAP). Our analysis delineates the chromosomal and regional distribution of ASE variants, identifies potential risk factors, and highlights variants showing differential ASE in AD versus Control samples. This work advances our understanding of the functional consequences of genetic variation in AD and offers novel insights into AD pathogenesis.

**RESEARCH IN CONTEXT**

1. **Systematic review**: The authors reviewed the literature using PubMed and conference proceedings. Prior studies demonstrated Allele‐specific expression's (ASE's) utility in identifying functional variants in Parkinson's disease and autism spectrum disorder. In Alzheimer's disease (AD), ASE analysis was limited to 33 known Genome‐wide association studies (GWAS) variants without genome‐wide scope. Relevant citations are appropriately cited.
2. **Interpretation**: Our genome‐wide ASE analysis across seven brain regions in 2,231 individuals identified 56,136 unique ASE variants enriched in imprinted chromosomal regions and known AD genes. We discovered 40 AD‐associated ASE variants whose affected genes were predominantly downregulated across multiple cell types, providing novel cell‐type–specific context for their roles in pathogenesis.
3. **Future directions**: Key questions include: (a) functional validation of AD‐associated ASE variants using CRISPR‐based approaches; (b) elucidating cis‐regulatory mechanisms driving differential ASE in AD; (c) evaluating whether ASE variants can serve as early biomarkers for risk stratification; and (d) determining how cell‐type–specific ASE changes contribute to disease progression.

## 2 METHODS

### 2.1 Variant calling and allele expression profile from RNA‐sequencing data

Raw RNA‐seq reads were aligned to the human reference genome hg19 using the STAR aligner (v2.3.0e) guided by Ensembl gene model GRCh37.70. The hg19 reference genome was used to ensure consistency with variant calling from whole‐genome sequencing data in the same cohorts which were publicly available from the AMP‐AD portal (see the Data Availability section). Except parameters specified below, default parameters were used for STAR (–chimSegmentMin 15 –chimJunctionOverhangMin 15 –outSAMstrandField intronMotif –outReadsUnmapped Fastx –outSAMtype BAM Unsorted –outSAMmode Full –outSAMunmapped Within). Default settings related to mismatches are listed as follows: –outFilterMismatchNmax 10 – outFilterMismatchNoverLmax 0.3 – outFilterMismatch NoverReadLmax 1.0. Variant calling in RNA‐seq data followed the GATK Best Practices v3.0 (https://gatkforums.broadinstitute.org/gatk/discussion/3891/calling‐variants‐in‐rnaseq). Starting from star‐aligned BAM files, Picard tools were used for adding read group information, sorting, marking duplicates and indexing. GATK (v3.4.0) tool SplitNCigarReads was applied to split reads into exon segments and trim any sequences overhanging into the intronic regions with parameters: ‐rf ReassignOneMappingQuality ‐RMQF 255 ‐RMQT 60 ‐U ALLOW_N_CIGAR_READS. GATK adjusted base‐quality score recalibration (BQSR) was called to detect systematic errors caused by the sequencer. After recalibration, single nucleotide polymorphisms (SNPs) and insertions/deletions (INDELs) were called jointly with the GATK HaplotypeCaller. A series of hard filters was applied to remove low‐quality variant sites using recommended parameters, including filtering clusters of at least 3 SNPs within a window of 35, Fisher Strand values (FS) > 30.0, or Qual By Depth values (QD) < 2.0. Finally, ASE quantification was conducted at bi‐allelic loci by the GATK tool ASEReadCounter with the following parameter settings (–U ALLOW_N_CIGAR_READS –minDepth 10 –minMappingQuality 10 –minBaseQuality 2 ‐drf DuplicateRead). Only heterozygous loci independently confirmed by DNA sequencing data were retained for downstream ASE analysis. Specifically, WGS‐derived genotypes from MSBB and ROSMAP, together with whole exome sequencing (WES)‐derived genotypes available for MSBB, were used to define heterozygous loci. RNA‐seq data were used to quantify reference‐ and alternative‐allele read counts at these genotype‐confirmed heterozygous loci.

### 2.2 Variant with allele expression across seven brain regions

**FIGURE 1:**
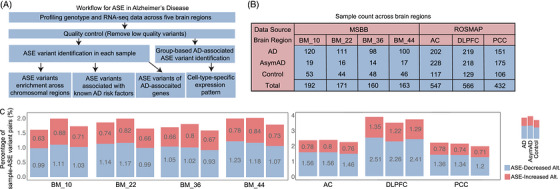
Workflow for the identification and analysis of ASE across brain regions. (A) Schematic overview of the study workflow. (B) Sample count for AD, AsymAD, and Control groups across brain regions, including BM_10, BM_22, BM_36 and BM_44 from MSBB, and AC, DLPFC and PCC from ROSMAP. (C) Percentage of sample‐ASE variant pairs identified by binomial test among AD, AsymAD, and Control samples across brain regions. Color represents the direction of ASE, with ASE‐Decreased Alt. defined as locus where alternative allele was mapped by significantly fewer RNA‐seq read than reference allele, and vice versa for ASE‐Increased Alt. AC, anterior caudate; AD, Alzheimer's disease; ASE, allele‐specific expression; DLPFC, dorsolateral prefrontal cortex; MSBB, Mount Sinai Brain Bank; PCC, posterior cingulate cortex; ROSMAP, Religious Orders Study/Memory and Aging Project.

Using the method described above, allele expression profiles were derived from RNA‐seq data generated in two independent AD cohorts. The first comprised samples from four brain regions provided by MSBB,^9^ including Brodman area 10 (frontal pole, BM10‐FP), Brodman area 22 (superior temporal gyrus, BM22‐STG), Brodman area 36 (parahippocampal gyrus, BM36‐PHG), and Brodman area 44 (inferior frontal gyrus, BM44‐IFG). The second comprised samples from three brain regions provided by ROSMAP,^15^ including anterior caudate (AC), dorsolateral prefrontal cortex (DLPFC), and posterior cingulate cortex (PCC). The profile consisted of 848,546 unique variants involving 15,006 protein‐coding genes from MSBB, 1,405,344 unique variants involving 16,406 protein‐coding genes from ROSMAP, and 592,990 overlapped unique variants involving 14,082 protein‐coding genes. All samples were categorized into Control, AsymAD, or AD, based on a rubric considering Consortium to Establish a Registry for Alzheimer's Disease (CERAD), Braak score, and Dementia status used in a previous study.^16^ Control was defined as samples with CERAD 0‐1 and Braak score 0–3 without dementia, where CERAD must be 0 if Braak score is 3. AsymAD was defined as samples with CERAD 1–3 and Braak score 3–6 without dementia. AD disease status was defined as samples with CERAD 2–3 and Braak score 3–6 with Dementia. Dementia was defined as Clinical Dementia Rating (CDR) score ≥ 1 or Mini‐Mental State Examination (MMSE) < 24.^17^ Samples that didn't meet the rubric or lacked required information were excluded. We further removed outlier samples in which more than 30% variants exhibited ASE, resulting in a final analytic set of 686 MSBB samples and 1,545 ROSMAP samples (Figure 1B and S1A). All participants signed an informed consent, an Anatomical Gift Act, and a repository consent.

### 2.3 ASE variant identification

To mitigate reference bias, we only retained variants with a higher mapping quality score (lowMAPQDepth/rawDepth ≤ 0.01) and a higher sample frequency (fraction > 0.2). Analysis was restricted to bi‐allelic heterozygous variants located at autosomal chromosomes to exclude the confounding effect caused by sex and X‐inactivation in biological females. To ensure adequate statistical power, we retained variants with a minimum read depth (reference allele count + alternate allele count) ≥ 10 in the RNA‐seq data.^18^ For each variant in each sample, a two‐sided binomial test was performed under the null hypothesis in a Bernoulli experiment with a success probability of 0.5 to determine whether a variant exhibited ASE. BH (Benjamini–Hochberg) was used to transform *p*‐value into false discovery rate (FDR), with significance defined as FDR ≤ 0.05.

### 2.4 Enrichment of ASE variant at chromosome regions

An empirical permutation‐based method was employed to identify chromosome or chromosomal band enriched for ASE variants among AD, AsymAD, or Control samples within each brain region. We randomly perturbed the ASE statuses of sample‐variants 100,000 times, where each time the number of significant sample‐variants per chromosome (band) was calculated. The enrichment *p*‐value for each chromosome (band) was defined as the fraction of ASE sample‐variant under random conditions that exceeded the real ones. Multiple cutoffs of BH‐corrected *p*‐value, 0.01, 0.05, and 0.15, were selected to define significance.

### 2.5 Association of ASE variant with age of death, *APOE* allele, and sex

The fraction of variants exhibiting ASE was calculated for each sample. Samples were subsequently classified into subgroups based on age of death, *APOE* genotype, or sex. For the age‐based analysis, AD samples were divided into two subgroups using the median age of death within AD samples as a threshold, which was directly applied to stratify Control samples. The Wilcoxon test was used to compare the fraction of ASE variants between the two subgroups, requiring a minimum of five samples per subgroup. Statistical significance was defined by *p*‐values less than 0.05 or 0.15.

### 2.6 AD‐associated ASE identification

For each brain region, a linear mixed effects model was employed to identify whether each variant exhibited differential ASE between AD and Control samples^19^ as follows:

$$\log\left(\mu \right)=\beta_{0}+\beta_{1}X_{1}+\beta_{2}X_{2}+\beta_{12}X_{1}X_{2}+\beta_{s}X_{s}$$

where μ denotes the read count mapped to one allele (reference or alternative) in a given sample, X_1_ denotes the allele type (reference or alternative), X_2_ denotes the sample disease status (AD or Control), X_1_X_2_ represents allelic imbalance difference and X_s_ denotes the sample ID. Fixed effects included *β_1_*, *β*_2_, and *β_12_*, while *β_s_* was treated as a random effect. This analysis was restricted to variants carried by at least five samples in both AD and Control groups. Variants exhibiting differential ASE need to fulfill three criteria: (i) significant differential ASE in a specific MSBB region with a *p*‐value less than 0.05; (ii) significant differential ASE in a specific ROSMAP region with a BH‐corrected *p*‐value less than 0.15; (iii) consistent direction of allelic imbalance difference (β_12_) in both a specific MSBB region and a specific ROSMAP region. For the identification of neuropathological variables associated with ASE, AD disease status (X_2_) was replaced by neuropathological measures (e.g., CERAD, Braak score) within the same model framework.

### 2.7 Single‐cell RNA‐seq analysis

We leveraged one of the most comprehensive single‐nucleus RNA sequencing datasets in AD by Mathys et al.^20^ This dataset includes 2.3 million nuclei isolated from the prefrontal cortex of 427 participants in the ROSMAP cohort, spanning a wide range of AD progression. We used preprocessed read count data from the original study, which identified and grouped nuclei into seven known cell types, including astrocytes (Ast), excitatory neuron (Ex), immune cells (Im), inhibitory neuron (In), oligodendrocytes (Oli), oligodendrocyte precursor cells (Opc), and vascular cells (Vas). The count matrix was normalized using the logNormalize method in the R package Seurat.^21^ Sample donors were classified into three disease statuses (Control, AsymAD, and AD) defined as above. To conduct cell‐type–specific differential expression analysis across disease statuses, we utilized the FindMarkers function in R package Seurat, with postmortem interval, sex, and age at death included as covariates. Genes with a Bonferroni‐corrected *p*‐value < 0.05 and |log2 (fold changes)| > 0.18 within one cell type were considered as significant differential expression. Visualizations of the cell‐type–specific gene expression and differential expression were produced using R package ggplot2.

## 3 RESULTS

### 3.1 Systematic identification of ASE variants across brain regions

We systematically identified ASE variants in 2,231 RNA‐sequencing samples spanning seven brain regions, including four cortical regions (Brodmann area 10 (BM_10), Brodmann area 22 (BM_22), Brodmann area 36 (BM_36), and Brodmann area 44 (BM_44)) from MSBB,^9^ and three regions (anterior caudate (AC), dorsolateral prefrontal cortex (DLPFC), posterior cingulate cortex (PCC)) from the ROSMAP cohort^15^ (Figure 1A‐B and S1A; Table S1 and S2; see the Methods section). Most samples, including 96.1% (659/686) in MSBB and 100% (1545/1545) in ROSMAP, were late‐onset AD older than 65 years old. We employed a rubric that considered CERAD, Braak score, and Dementia status^16^ (see the Methods section) to classify samples into three groups: AD, AsymAD, and Control, with group‐specific sample size detailed in **Figure** **1B**.

Utilizing a robust ASE detection pipeline, bi‐allelic heterozygous variants of autosomal chromosomes that passed stringent quality control were analyzed in each sample using a binomial test (see the Methods section). This analysis identified 56,136 unique ASE variants across 10,416 protein‐coding genes (Figure 1C and S1B). Despite the extensive variant catalog analyzed, ASE was observed at only a small fraction of heterozygous loci, accounting for less than 2% in MSBB samples and less than 4% in ROSMAP samples, where the larger sample size of ROSMAP conferred greater statistical power (Figure 1C and S1B; Table S3). The frequency distribution of ASE variants followed a power‐law pattern: a small subset of variants recurred across samples, while the majority were detected in a limited number of samples (Figure S1C). Across brain regions, we observed a trend of more variants showing decreased expression of the alternative allele, abbreviated as ASE‐Decreased Alt. (Figure 1C; Table S3).

To evaluate whether ASE identifications were influenced by reference bias, where there may be reduced mapping of RNA‐seq reads onto alternative alleles,^13^, ^18^ ASE variants were stratified by genomic location. ASE‐Decreased Alt. were significantly enriched in exonic regions compared to non‐exonic regions (*p* < 0.01, Figure S2A), consistent with the known effects of coding variants on transcript stability through mechanisms like nonsense‐mediated decay (NMD). In contrast, non‐exonic ASE variants demonstrated a nearly 50% split of variants between ASE‐Decreased Alt. and ASE‐Increased Alt. (Figure S2A), indicating minimal reference bias in our analysis pipeline. We also investigated ASE variants within known human imprinted genes,^22^ which exhibit monoallelic expression due to parent‐of‐origin effects. Imprinted genes harbored a significantly higher frequency of ASE variants compared to other genes (*p* < 0.01, Figure S2B), further demonstrating the pipeline's sensitivity in capturing biologically meaningful ASE patterns.

### 3.2 Enrichment of ASE variants across chromosomal regions

**FIGURE 2:**
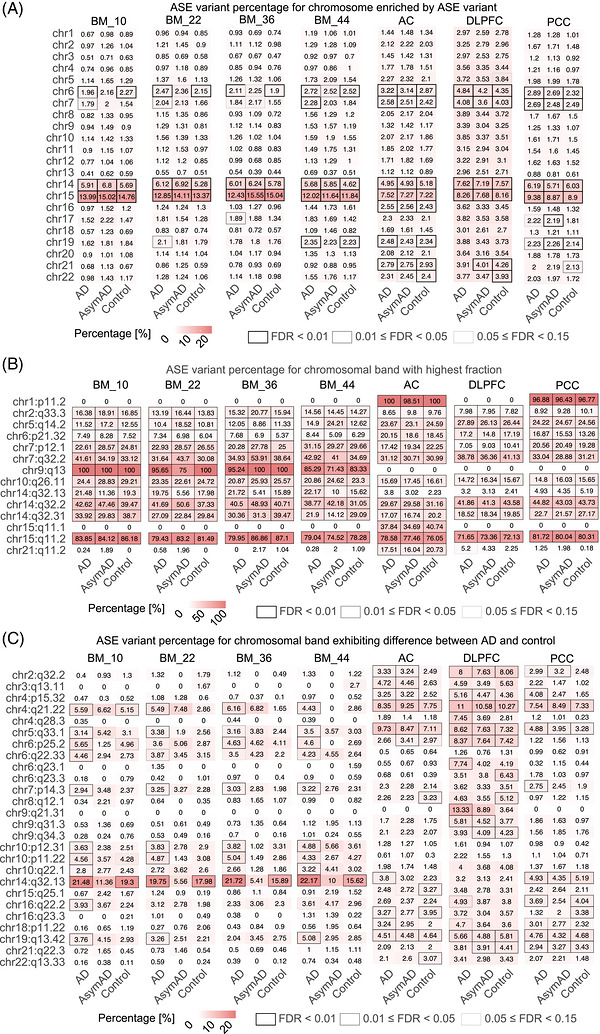
Enrichment of ASE variants across chromosomal regions. Each cell represents the percentage of ASE variants for 22 autosomal chromosomes (A), chromosomal bands with highest ASE variant fraction (B), and chromosomal bands exhibiting different patterns of ASE variants enrichment between AD and Control samples (C). *p*‐Value was calculated by permutating ASE variant labels within samples of each brain region. Benjamini–Hochberg was used to adjust the *p*‐value into FDR, denoted by the boxes. AD, Alzheimer's disease; ASE, allele‐specific expression; FDR, false discovery rate.

To investigate the genomic distribution of ASE variants, we performed a systematic chromosome‐level permutation analysis (see the Methods section) and observed significant enrichment of ASE variants on chromosomes 6, 14, and 15 in both AD and non‐AD samples across all brain regions (Figure 2A and S3A). These three chromosomes harbor established imprinted genes that can lead to clinical pathology if uniparental disomy occurs.^23^ In contrast, several other chromosomes, including 7, 17, and 19, showed sporadic enrichment, primarily limited to subsets of AD samples. In the ROSMAP cohort, ASE variants on chromosomes 21 and 22 were enriched in Control samples. All remaining chromosomes displayed an overall depletion of ASE variants across all brain regions (Figure 2A and S3A).

To achieve finer genomic resolution, we performed the same permutation analysis by each chromosomal band (Table S4; see the Methods section**)**. Notably, nearly all variants located within the chr9q13 exhibited enrichment for ASE across the four MSBB brain regions. This pattern was not observed in the ROSMAP, due to variants at this region with lower sample frequency or mapping quality (Figure 2B and S3B). ASE variants were enriched at chr14q32 and chr15q11 across all brain regions (Figure 2B and S3B), aligned with prior findings in ASD brain region analysis,^13^ Prader–Willi syndrome and Angelman syndrome.^24^ Additional ASE‐enriched chromosomal bands included chr2q33, chr7p12, chr7q32, and chr10q26 (Figure 2B and S3B). Furthermore, distinct patterns of ASE variant enrichment between AD and Control samples were observed at several chromosomal bands, including chr4q21.22 and chr7p14.3 (Figure 2C and S3C). The most pronounced differences was observed at chr4q21.22 in BM_36 (MSBB), and at chr4q28.3 and chr9q21.31 in DLPFC (ROSMAP), with increases of 4.51%, 4.64%, and 9.69% in ASE variant fraction in AD samples relative to Control samples (FDR < 0.01, Figure 2C and S3C). These findings established a comprehensive enrichment landscape of ASE variants, highlighting specific chromosomal regions with potentially dysregulated ASE in AD pathogenesis.

### 3.3 Association of ASE variant fraction with age of death, *APOE* allele, and sex

**FIGURE 3:**
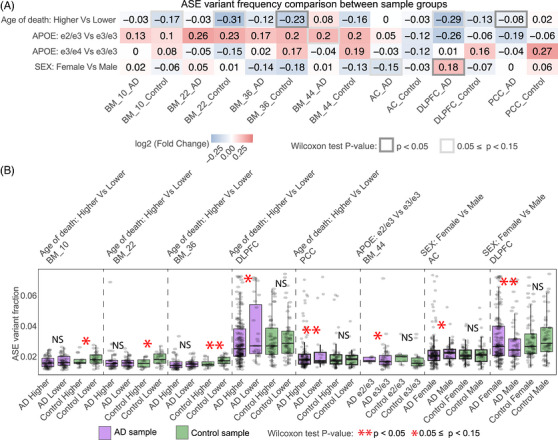
Association of ASE variant frequency with age of death, *APOE* genotype and sex. (A) Comparison of ASE variant frequency between samples stratified by AD risk factors (age of death, *APOE* genotype, and sex) for AD and Control groups across brain regions. Each cell represents log2 scale of fold change of median ASE variant fraction compared between risk factor levels across AD disease status and brain regions. *p*‐Value was calculated by Wilcoxon test and denoted with boxes. (B) Fraction of ASE variants showing suggestive or significant associations in the comparison analysis from (A). AD, Alzheimer's disease; ASE, allele‐specific expression; APOE, apolipoprotein E.

We next evaluated the relationship between each sample's overall ASE variant fraction and known AD risk factors, including age of death, *APOE* allele status, and sex,^25^, ^26^ stratified by AD disease status (see the Methods section). This analysis seeks to identify clinical factors that may affect ASE dysregulation across all variants in a sample on a global scale. Interestingly, our analysis revealed that higher age of death was suggestively or significantly associated with a lower frequency of ASE variants across most brain regions, regardless of AD disease (Figure 3). We next examined the influence of *APOE* genotype on overall ASE fraction across samples. Compared to *APOE* e3 homozygotes, samples carrying the *APOE* e2 allele exhibited an elevated frequency of ASE variants in both AD and Control samples across all MSBB brain regions, whereas an inverse trend was observed in DLPFC and PCC of ROSMAP, albeit only BM_44 AD samples showed a suggestive association (Figure 3). When comparing *APOE* e4 carriers to e3 homozygotes, a trend of higher ASE variant frequency was observed among the Control samples across five brain regions, BM_10, BM_36, BM_44, DLPFC, and PCC (Figure 3). Regarding sex difference, female AD samples exhibited a slightly higher ASE frequency compared to male AD samples in four brain regions, with this trend reaching significance in DLPFC, whereas an opposite trend was observed in Control samples albeit not statistically significant (Figure 3). These analyses highlight risk factors that may regulate global ASE at the individual level, but require validation in larger cohorts.

### 3.4 ASE variants implicated in AD GWAS genes

**FIGURE 4:**
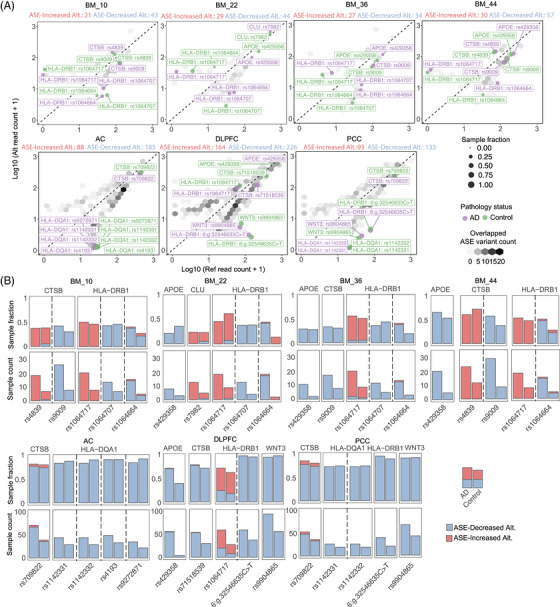
ASE variants within AD‐associated genes across brain regions. (A) Density plot for all ASE variants within AD‐associated genes. Each dot represents the mean log (read count + 1) among carriers from specific cohort for a given ASE variant. The top five ASE variants with highest fraction in all the carriers among AD samples were labeled out in the point plot. Color represents sample AD disease, AD, or Control. Point size denotes the carrier fraction of the ASE variants. The number of ASE‐Increased Alt. or ASE‐Decreased Alt. were marked above the plot for each brain region. (B) Carrier fraction (upper) and count (lower) of AD or Control samples for the top five ASE variants with highest fraction in all the carriers among AD samples from (A). Color represents the direction of ASE, with ASE‐Decreased Alt. defined as locus where alternative allele mapped by significantly fewer RNA‐seq read than reference allele, and vice versa for ASE‐Increased Alt. AD, Alzheimer's disease; ASE, allele‐specific expression.

ASE can serve as a powerful instrument for connecting GWAS genetic loci to downstream expression changes and mechanisms. We examined ASE variants across AD‐associated genes curated from previous GWAS or exome‐based studies.^6^, ^7^, ^8^ Across all brain regions, a higher fraction of ASE variants exhibited decreased expression of the alternative allele among these AD‐associated genes (Figure 4A and S4A). We next focused on distinct ASE patterns of variants with the highest ASE frequency in the AD samples (Figure 4B and S4B; Table S5). Notably, our analysis identified the variant rs429358 that tags *APOE2/3/4*, which affects cysteine (C) and arginine (R) residues at positions 130, with a high fraction of ASE‐Decreased Alt. in AD samples across most brain regions (Figure 4B and S4B). The variant rs1064664 (*HLA‐DRB1* p.Y61H) also showed preference for and higher frequencies of ASE‐Decreased Alt. in AD samples compared to Control samples across all four brain regions from MSBB (Figure 4B and S4B). Other variants with overall higher fractions of ASE‐Decreased Alt. included rs1064707 at 3′‐untranslated region (UTR) or noncoding exon of *HLA‐DRB1*, rs9009 at 3′‐UTR or noncoding exon or intron of *CTSB* across MSBB brain regions, and rs9904865 at noncoding exon or intron of *WNT3* in DLPFC and PCC, and rs1142331 (p.G84C) / rs1142332 (p.G84V) at coding exon or noncoding exon of HLA‐DQA1 in AC and PCC (Figure 4B and S4B). For *CLU*, which had genetic loci associated with AD,^6^, ^7^ synonymous variant rs7982 (p.H229/263/274/315H, depending on the isoform), demonstrated ASE‐Increased Alt. in BM_22 (Figure 4B and S4B). Additional variants showing ASE‐Increased Alt. include rs1064717 at the noncoding exon of *HLA‐DRB1* in most brain regions and rs4839 at 3′‐UTR or noncoding exon or intron of *CTSB* in BM_10 and BM_44 (Figure 4B and S4B), extending prior findings of these genes’ involvement in AD. These observations identify specific variants covered by RNA‐seq that may help bridge GWAS findings to downstream changes in transcript expression.

### 3.5 AD‐associated ASE variants and cell‐type–specific expression of affected genes

To identify variants showing ASE associated with AD disease status, we employed a linear mixed effects model adjusting for individual‐level random effects^19^ (see the Methods section). This analysis identified 40 unique AD‐associated ASE variants spanning 35 protein‐coding genes displaying differential ASE between AD and Control samples in both MSBB and ROSMAP cohorts, where the majority (75%, 30/40) exhibited a larger allele imbalance in AD samples compared with Control samples (Figure S5A, see the Methods section).

We further examined the cell‐type–specific expression pattern of genes harboring these AD‐associated ASE variants. We obtained a postmortem brain single‐cell RNA‐seq dataset from the ROSMAP cohort,^27^ where the cells were clustered into seven major cell types, including astrocyte (Ast), excitatory neurons (Ex), immune cells (Im), inhibitory neuron (In), oligodendrocyte (Oli), oligodendrocyte progenitor cell (Opc), and vasculature cells (Vas) (Table S6). We stratified cells of each cell type by their originating sample's AD disease status and examined expression changes between AD statuses for the 32 genes profiled in the single‐cell RNA‐seq dataset (see the Methods section).

**FIGURE 5:**
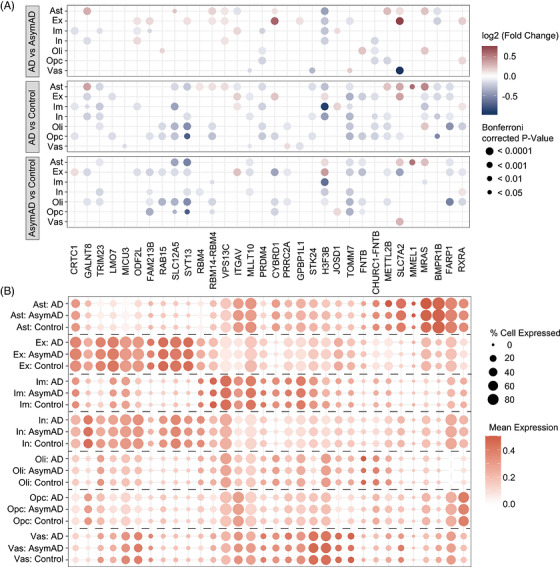
Cell‐type–specific expression analysis of genes affected by variants exhibiting differential ASE between AD and Control samples, shown at Figure S5A. Cell‐type–specific differential expression information (A) and expression levels (B) for genes harboring variants that display differential expression between AD disease status in at least one cell type. Statistical significance was calculated by Wilcoxon rank sum test, with significance defined as |log2 (fold change)| > 0.18 and Bonferroni‐corrected *p*‐value < 0.05. AD, Alzheimer's disease; ASE, allele‐specific expression.

Our analysis revealed a predominant downregulated pattern among the 32 genes in AD or AsymAD cells compared to Control cells (|log2 (Fold change)| > 0.18 & Bonferroni‐corrected *p*‐value < 0.05), mostly prominent in Ex, In, Oli, and Opc, with a subset of genes displaying upregulation in Ast (Figure 5). Notably, the magnitude of transcriptional change was substantially greater and consistent in the AD or AsymAD versus control comparisons than in the AD versus AsymAD comparison, suggesting that these transcriptional alterations are established early in the disease course, likely prior to the onset of clinical symptoms.

Ex and In neurons exhibited broad expression of genes such as SLC12A5, SYT13, CRTC1, and LMO7, with largely consistent patterns of downregulations in AD samples (Figure 5). In particular, *SLC12A5* and *SYT13* were significantly downregulated in both AD and AsymAD cells relative to control cells across multiple cell types (Figure 5). Reduced expression of *SLC12A5* is known to trigger apoptotic pathway leading to neuronal cell death, a hallmark of AD,^28^, ^29^ while diminished *SYT13* expression has been shown to confer selective vulnerability to motor neuron diseases.^30^ Our findings delineate the specific cellular context where *SLC12A5* and *SYT13* may contribute to AD progression. *TRIM23*, a gene associated with autophagy or apoptosis,^31^, ^32^, ^33^ was also downregulated across three cell types (Ast, In, and Oli) in both AD and AsymAD cells relative to Control cells. Another example was TOMM7, encoding translocase of the outer mitochondrial membrane. Its expression was downregulated across five cell types (Ast, Ex, In, Oli, Opc) in both AD/AsymAD cell groups relative to Control cells (Figure 5). Reduced TOMM7 expression has been reported in AD tissue^34^ and implicated with other neurodegeneration conditions.^35^ In contrast, *VPS13C*, a gene linked to Parkinson's disease via perturbing mitochondrial function,^36^, ^37^ was upregulated in Ast in the AD versus control comparison (Figure 5). *LMO7*, one of the top 10 hub genes in an AD‐associated protein‐protein interaction network,^38^ exhibited downregulated expression in both Ex and Opc in the AD versus control comparison (Figure 5).

We additionally applied the linear mixed effects model to two neuropathological variables (CERAD or Braak score) and identified neuropathology‐associated ASE variants (Table S7). The direction of allele imbalance for these neuropathology‐associated ASE variants was fully concordant with the prediction derived from the binary disease classification‐based model; however, only one ASE variant achieving significance in both the classification‐based and Braak score‐based models for BM_22/DLPFC region pair (Table S7): rs12463 at STK24, a gene that interacts with established AD genes^39^ and is associated with brain development.^40^ This limited overlap in statistical significance despite full directional concordance likely reflects differences in power across the modeling approaches rather than biological discordance. These analyses also implicated additional genes with reported association with AD. For example, knockdown of GRIN2A, which carries a CERAD‐associated ASE variant rs62034910, has been shown to aggravate memory and cognitive deficits,^41^ a core feature of AD. Decreased expression of PSMA7, harboring Braak score‐associated ASE variant rs1135961, has been associated with AD progression.^42^ Furthermore, a systematic correlation assessment revealed that the interaction coefficients derived from CERAD or Braak score were highly correlated with those obtained from the binary disease classification‐based model, and the correlation between the classification‐based and either measure exceeded the correlation between CERAD and Braak score (Figure S5B). We also performed cell‐type–specific differential expression analysis for neuropathology‐associated ASE variant genes (Figure S5C‐F). In summary, this analysis identified several AD‐associated ASE variants and provided cell type context for how these genes may contribute to AD pathogenesis and progression.

## 4 DISCUSSION

Our study provides, to our knowledge, the first systematic characterization of ASE across multiple brain regions in AD, revealing the regulatory complexity of genetic variants at the mRNA level. By leveraging large RNA‐sequencing datasets encompassing seven brain regions from two independent cohorts, we identified 56,136 ASE variants across 10,416 protein‐coding genes. Despite analyzing a comprehensive catalog of bi‐allelic heterozygous variants, significant ASE events were relatively rare, occurring in less than 2%‐4% of variants. This aligns with previous reports suggesting that ASE is likely constrained to specific loci with functional relevance.

A key finding in our study is the enrichment of ASE variants in specific genomic regions, notably chromosomes 6, 14, and 15, which harbor established imprinted genes.^23^ This enrichment remained consistent across both AD and non‐AD samples, supporting the robustness of our analytical approach. Additional chromosomal bands, including chr14q32 and chr15q11, were enriched for ASE, aligning with prior studies linking these regions to neurodevelopmental disorders, such as ASD brain region analysis,^13^ Prader–Willi syndrome, and Angelman syndrome.^24^ The observed depletion of ASE variants in several other chromosomal regions, including those that showed unique enrichments in AD, underscores the potential influence of chromatin structure, transcription factor occupancy, and differential regulatory mechanisms in shaping allele‐specific transcriptional activity in the brain.

The association between ASE frequency and key AD risk factors highlights the potential clinical implications of our findings. Specifically, our analysis revealed a negative correlation between age of death and ASE variant frequency, suggesting that ASE may diminish with aging irrespective of AD disease. The differential effects of *APOE* alleles on ASE frequency further emphasize the genetic regulation of allele‐specific transcription in AD, with *APOE* e2 carriers generally exhibiting higher ASE frequencies in MSBB cohort, whereas *APOE* e4 carriers displayed an increasing ASE trend only in Control samples across most brain regions. These observations suggest that *APOE* genotype may modulate RNA stability or processing, warranting further investigation. Additionally, sex‐stratified analyses revealed that female AD samples had a significantly higher ASE frequency in DLPFC, a sex‐dependent effect that is noteworthy given the known sex differences in AD pathogenesis.^43^ However, these clinical associations with ASE require larger sample sizes to establish robust statistical significance validation. We also noted that findings differed between the MSBB and ROSMAP cohorts, which may reflect differences in brain region samples, sequencing depths, where MSBB tends to include a higher proportion of subjects with advanced Alzheimer's pathology and greater ethnic diversity compared to ROSMAP. To ensure robustness, we therefore focused on the concordant findings across the two cohorts in our downstream differential ASE analysis.

Focusing on AD‐associated genes found by GWAS or exome studies,^6^, ^7^, ^8^ we observed a pronounced bias toward ASE‐Decreased Alt. variants. The *APOE* locus included a significant ASE variant rs429358 (p.C130/156R), that tag *APOE2/3/4*. Additionally, our findings implicated additional exonic ASE variants, including rs1064664 (*HLA‐DRB1* p.Y61H) and rs7982 (*CLU* p.H229/263/274/315H), that can help detect potential regulatory mechanisms beyond canonical GWAS associations. These variants could serve as biomarkers or functional candidates that link GWAS findings to downstream transcriptional consequences. To our knowledge, the study by He et al.^14^ is the only other study that focused on ASE using AD samples, albeit to a considerably more limited extent. Whereas their analysis focused on ASE quantitative trait loci (aseQTL) associated with 33 AD‐associated GWAS variants, our study systematically examined genome‐wide ASE of all heterozygous exonic variants, enabling discovery beyond known AD GWAS genes.

Our differential ASE analysis identified 40 unique AD‐associated ASE variants across 35 protein‐coding genes in both MSBB and ROSMAP cohorts, highlighting *cis*‐regulatory variation that may contribute to AD pathogenesis. By integrating single‐cell RNA‐seq data, we observed that 32 of these genes exhibited predominant downregulation pattern in AD across multiple cell types. In addition to the two genes (*SLC12A5* and *TRIM23*) implicated in neuronal cell death, which is an established AD hallmark,^28^, ^29^, ^31^, ^32^, ^33^ *BMPR1B* is a member of the bone morphogenetic protein (BMP) family that directly influences cell death^44^, ^45^ and is also implicated in neurogenesis.^46^ Additionally, we found that *TOMM7* and *VPS13C*, two genes linked to mitochondrial dysfunction in AD or other neurodegenerative disorder,^34^, ^35^ displayed reverse dysregulated expression in multiple cell types. Several genes with AD‐associated ASE variants have been functionally validated in murine experiments, such as CRTC1, MMEL1, MICU3, and SLC12A5, in traits related to AD. Dysfunction of the CREB coactivator CRTC1 impaired synaptic plasticity and memory, one core feature of AD progression.^47^, ^48^, ^49^ Gene knockout and transgenic models have proved that MMEL1 encodes a protease that degrades amyloid‐β peptides, and its altered activity exacerbates plaque accumulation.^50^ MICU3, a mitochondrial calcium uptake regulator, has been implicated in cerebral amyloid angiopathy,^51^ a contributor of AD, which complements our finding that other mitochondria‐associated ASE variant genes (TOMM7, VPS13C) are dysregulated across multiple cell types. Loss of function of the potassium‐chloride cotransporter SLC12A5 triggers neuronal apoptosis,^28^, ^29^ consistent with its marked downregulation across cell types in both AD and AsymAD in our single‐cell analyses. Together, these experimental and computational lines of evidence highlight that ASE may be a functionally relevant mechanism affecting genes spanning synaptic dysfunction, amyloid proteostasis, mitochondrial impairment, and neuronal survival in AD progression.

Our study has several limitations. First, although we controlled for reference allele mapping bias and technical artifacts, additional validation using orthogonal technologies (e.g., long‐read sequencing or allele‐specific reporter assays) is needed to confirm ASE effects. Second, differences in sequencing depth and RNA integrity across brain regions may have influenced ASE detection sensitivity. Third, our findings are limited to *post mortem* brain tissues, and future studies incorporating single‐cell resolution or longitudinal datasets may provide further insights into the temporal and cellular specificity of ASE regulation in AD. Finally, our human‐cohort findings, while using ASE as a powerful instrument, require experimental validation to establish the causal role of an individual gene or variant in AD pathogenesis.

In summary, this study systematically maps ASE landscapes across seven brain regions, revealing distinct patterns of allele‐specific regulation associated with genomic architecture, AD risk factors, and disease‐relevant genes. Our findings provide a resource for understanding transcriptional dysregulation in AD and nominate novel targets for functional follow‐up and therapeutic development.

## AUTHOR CONTRIBUTIONS

Kuan‐lin Huang, Minghui Wang and Zishan Wang conceived the research and designed the analysis. Minghui Wang curated the dataset from MSBB and ROSMAP and conducted genomic analyses to extract all ASE read counts. Zishan Wang performed all ASE association analysis. Delowar Hossain and Judy Wang conducted the single‐cell analyses. Zishan Wang, Varun R. Subramaniam, Kuan‐lin Huang, and Minghui Wang wrote the manuscript. Kuan‐lin Huang and Minghui Wang supervised the study. All the authors read, edited, and approved the manuscript.

## CONFLICT OF INTEREST STATEMENT

All authors declare that they have no competing interests. Author disclosures are available in the Supporting Information.

## CONSENT STATEMENT

The datasets included in this study were from publicly available datasets, namely MSBB and ROSMAP. Relevant ethical approval was obtained for both MSBB and ROSMAP, and informed consent was received from all participants prior to participation. Therefore, no additional ethical approval was required for this study.

## SOFTWARE AVAILABILITY

Code scripts to reproduce the analyses are deposited at Github: https://github.com/WangZishan/ADASE

## Supporting information




Supporting Information

Supporting Information

Supporting Information

Supporting Information

Supporting Information

Supporting Information

Supporting Information

## Data Availability

The multi‐omics datasets of MSBB and ROSMAP are available via the AD Knowledge Portal (https://adknowledgeportal.org). The AD Knowledge Portal is a platform for accessing data, analyses, and tools generated by the Accelerating Medicines Partnership (AMP‐AD) Target Discovery Program and other National Institute on Aging (NIA)‐supported programs to enable open‐science practices and accelerate translational learning. The data, analyses and tools are shared early in the research cycle without a publication embargo on secondary use. Data is available for general research use according to the following requirements for data access and data attribution (https://adknowledgeportal.synapse.org/Data%20Access). The MSBB bulk tissue data is available at https://www.synapse.org/Synapse:syn3159438 and the ROSMAP bulk tissue data is available at https://www.synapse.org/Synapse:syn3219045. The significant ASE variants identified by binomial test is available at figshare platform https://doi.org/10.6084/m9.figshare.31549300. The ROSMAP snRNA‐seq data is available at https://www.synapse.org/Synapse:syn52293417.
