# Supplementary figures and images for: Allele specific expression in Alzheimer's disease
Source: Alzheimers Dement. 2026 Jun 11;22(6):e71558. doi: 10.1002/alz.71558 (PMC13254825; doi:10.1002/alz.71558)

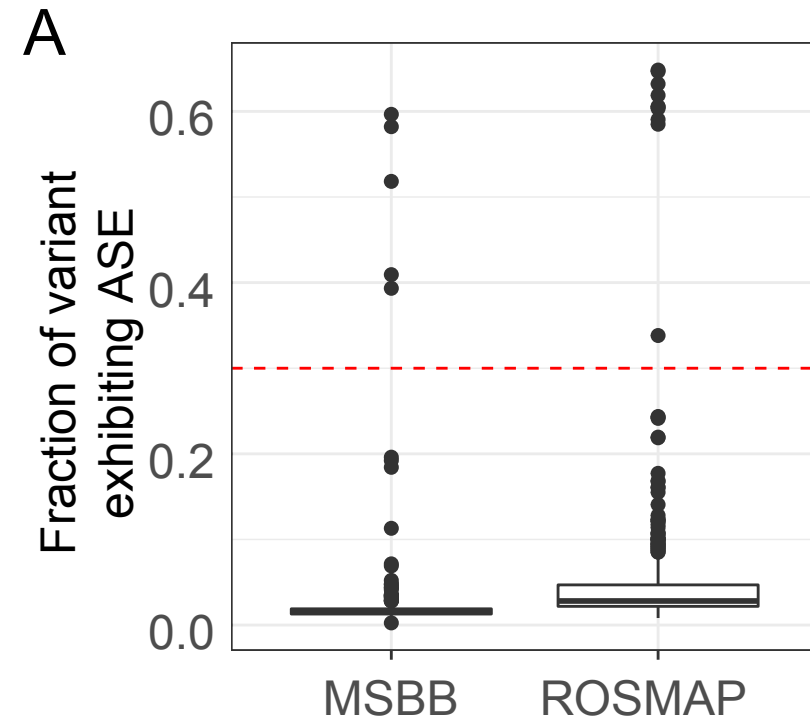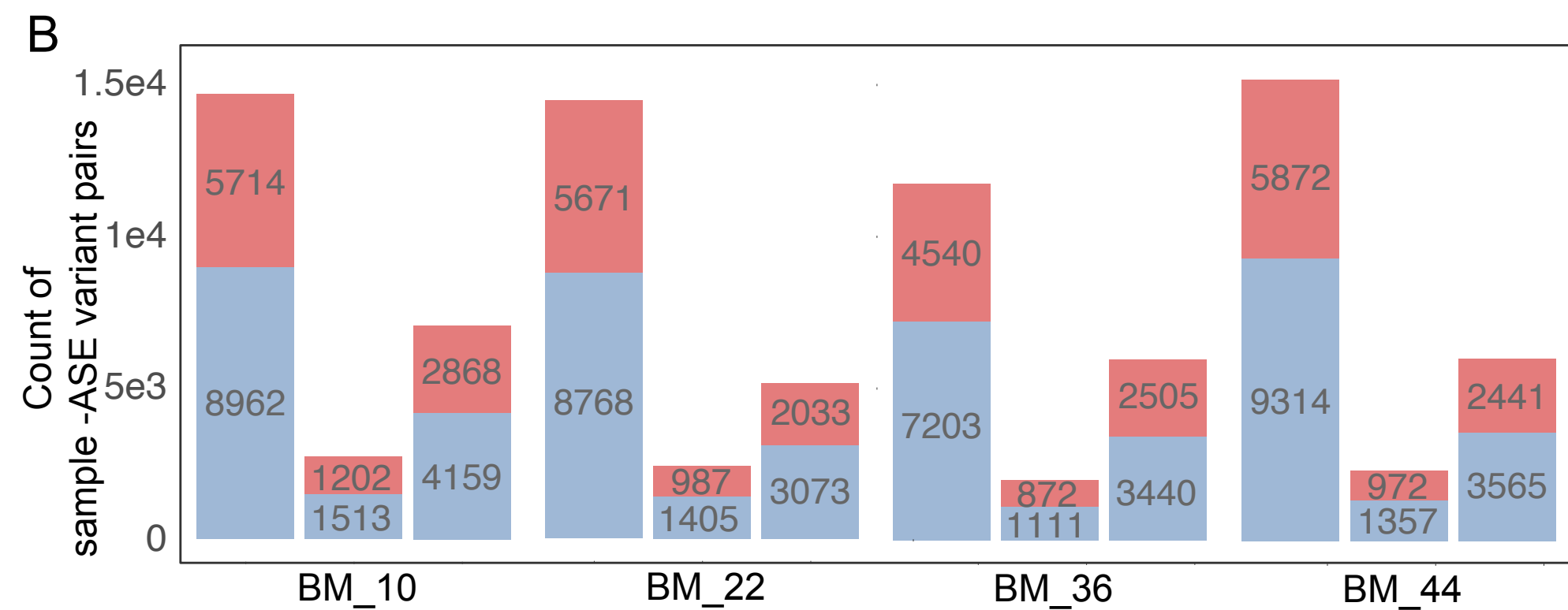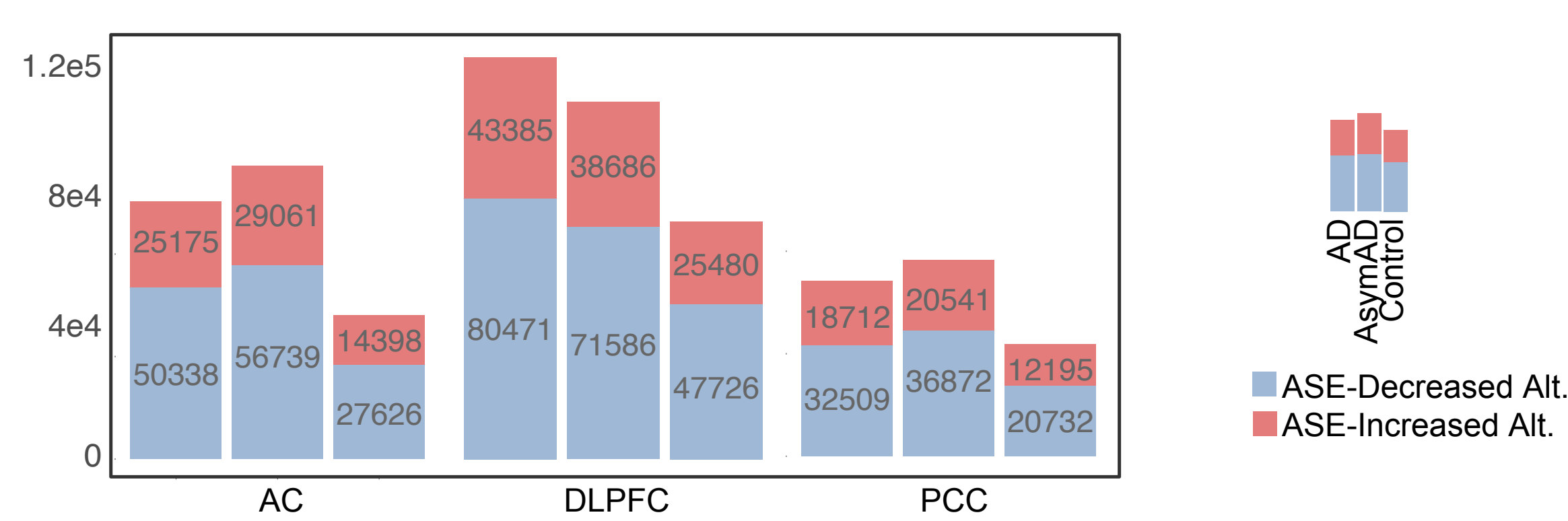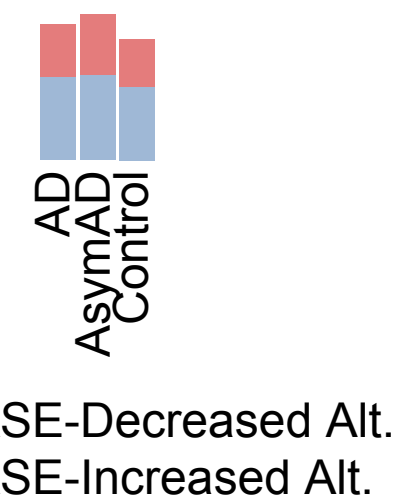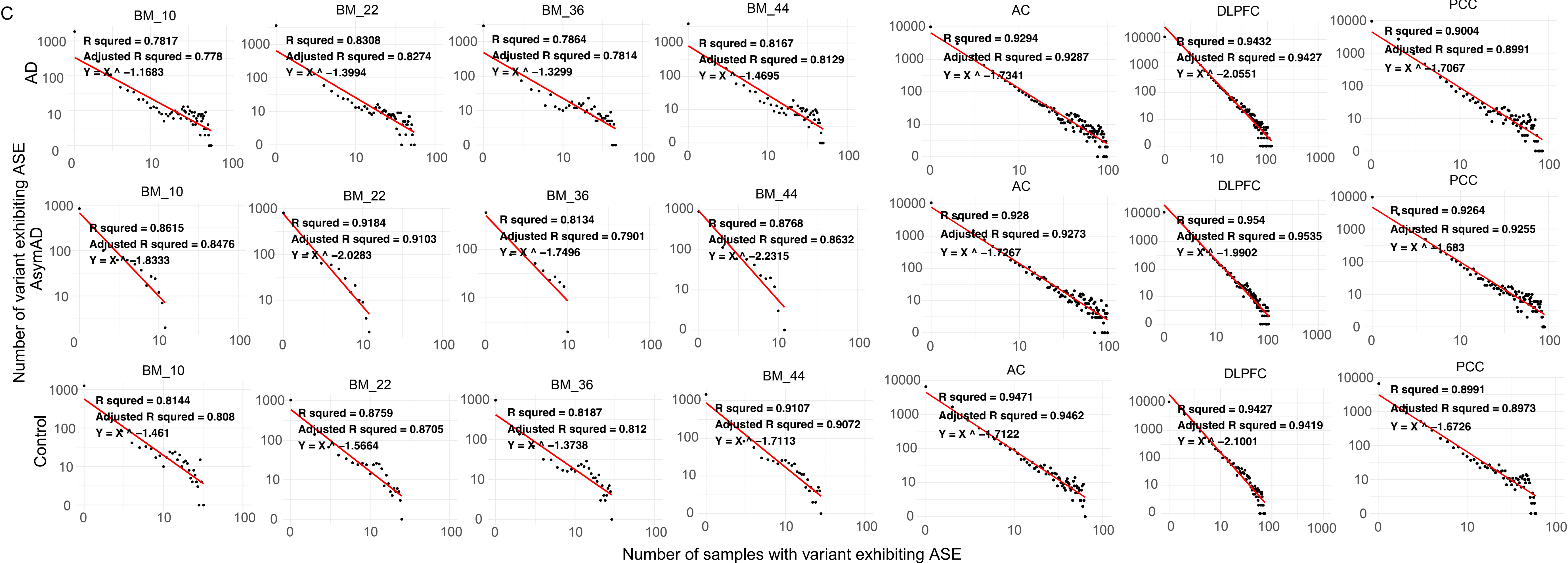

Supplement: Supplementary file 1 — Supporting Information [file ALZ-22-e71558-s005.pdf]

A

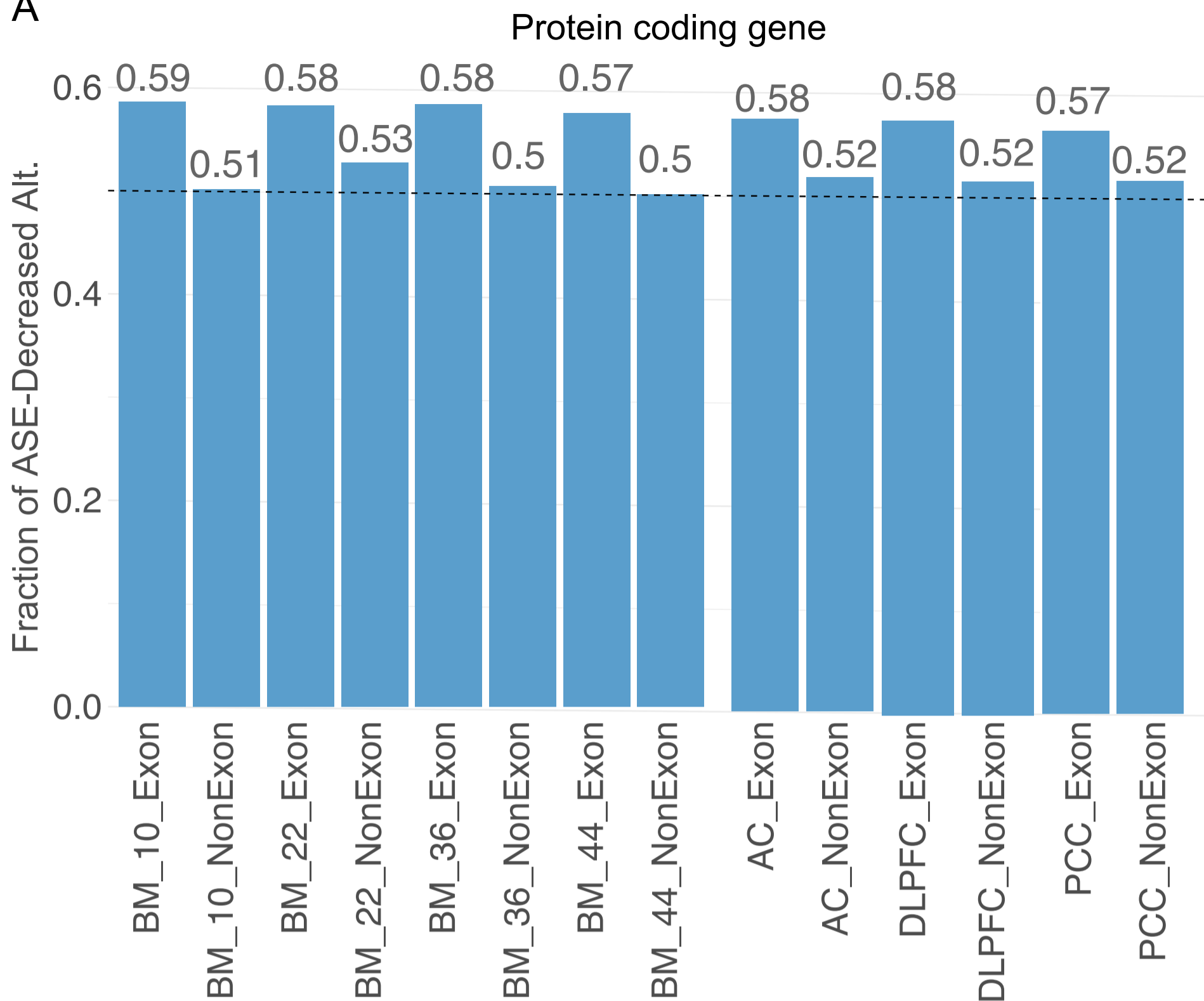

B

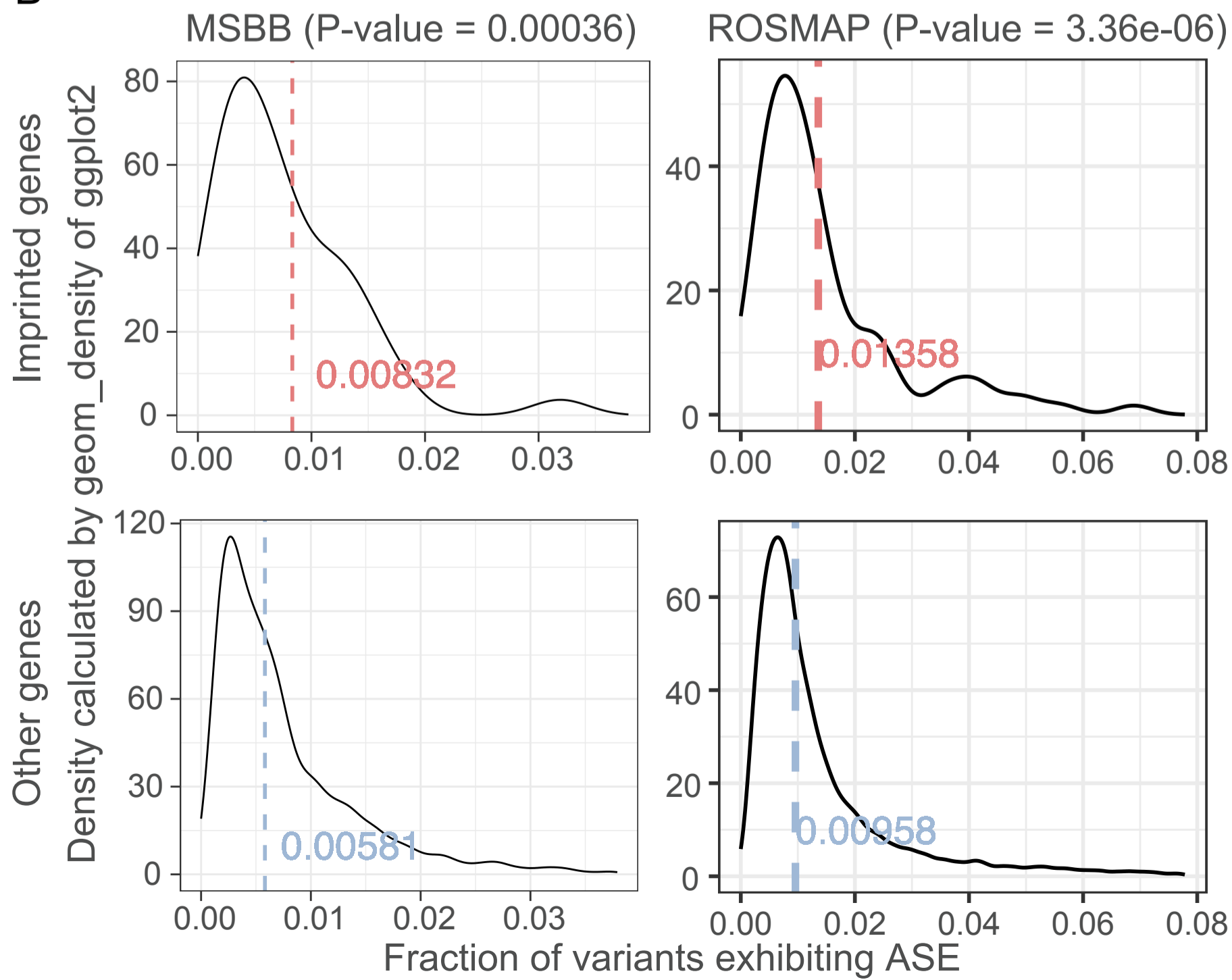

Supplement: Supplementary file 2 — Supporting Information [file ALZ-22-e71558-s001.pdf]

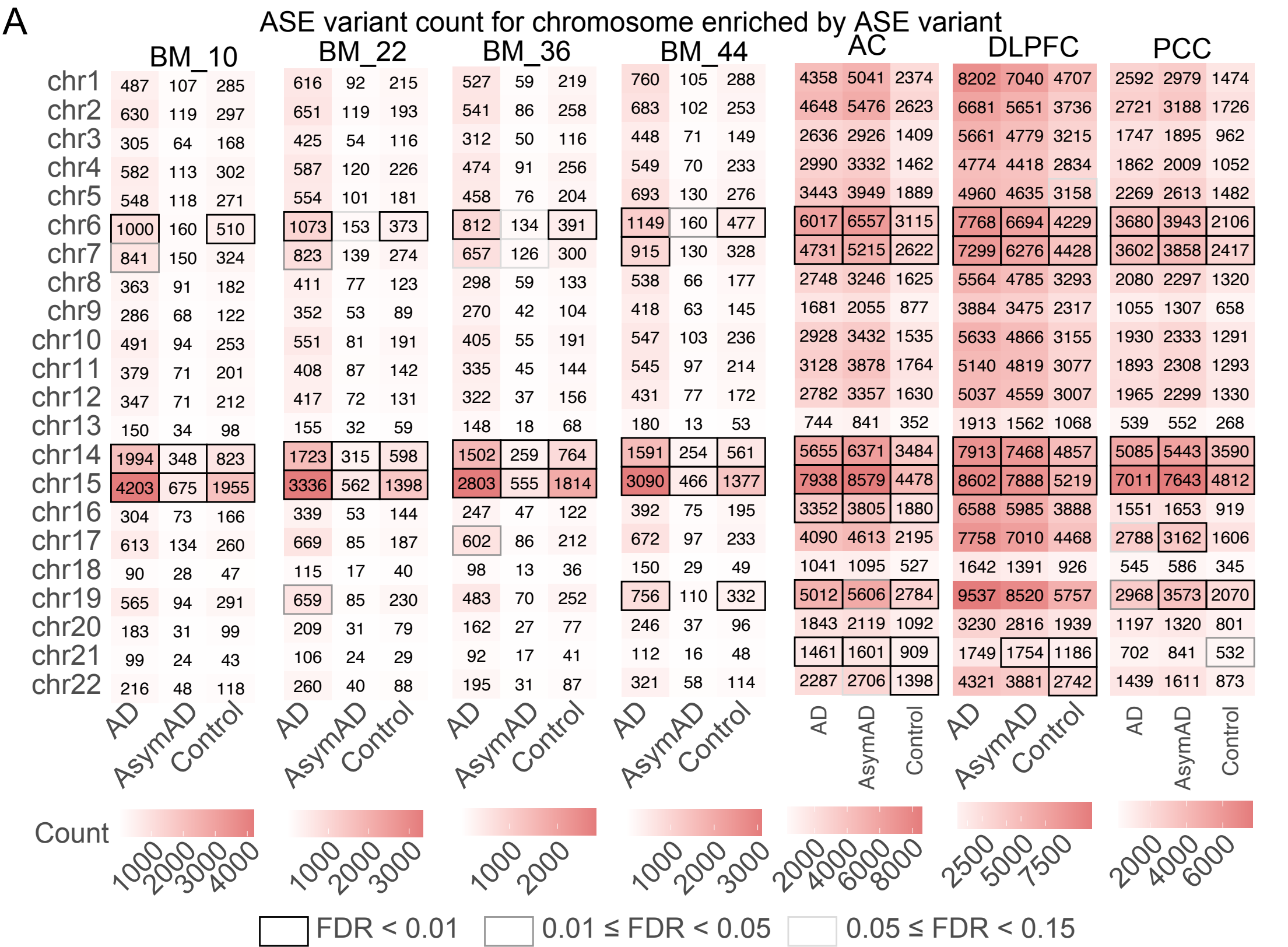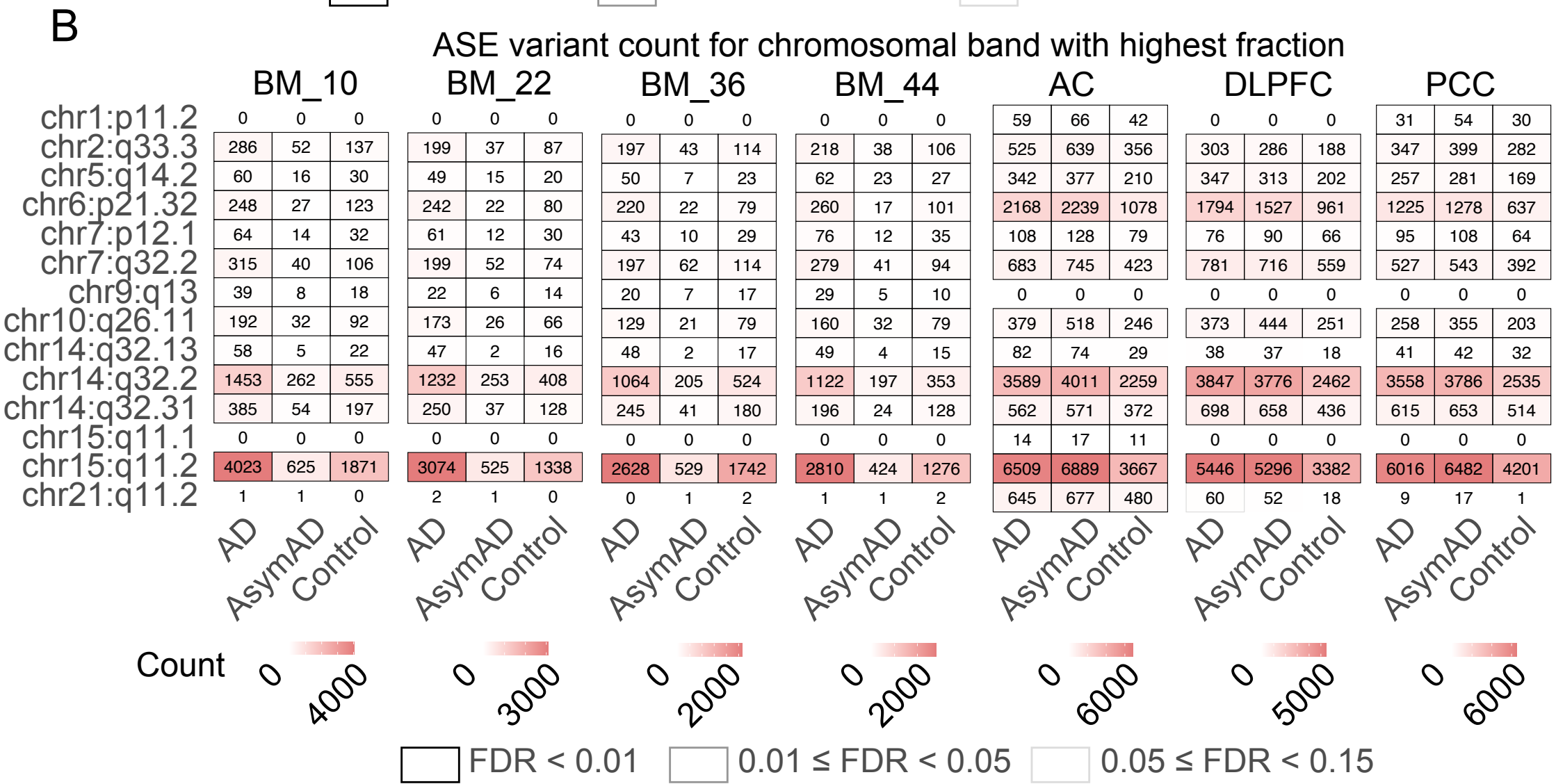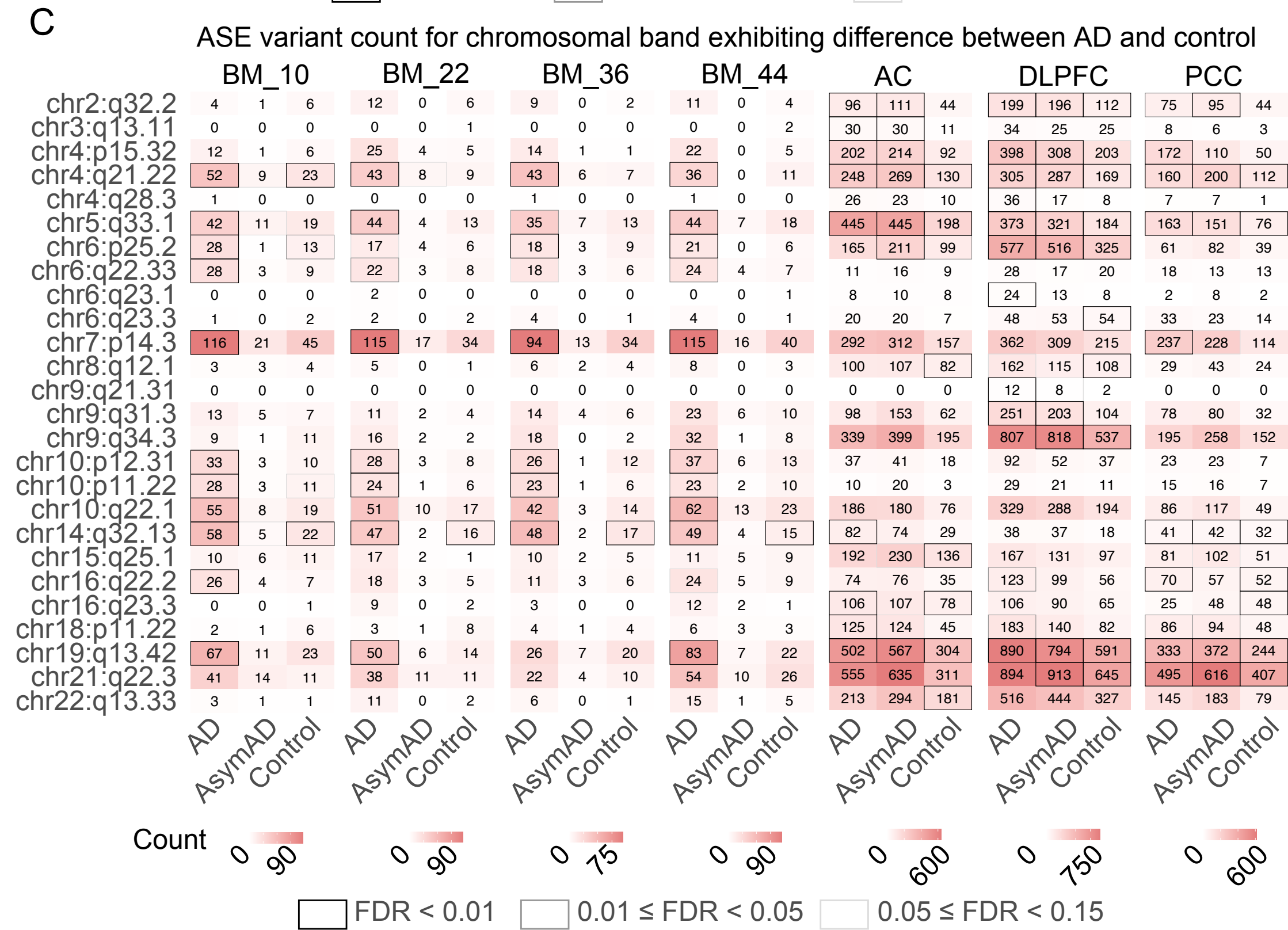

Supplement: Supplementary file 3 — Supporting Information [file ALZ-22-e71558-s004.pdf]

A

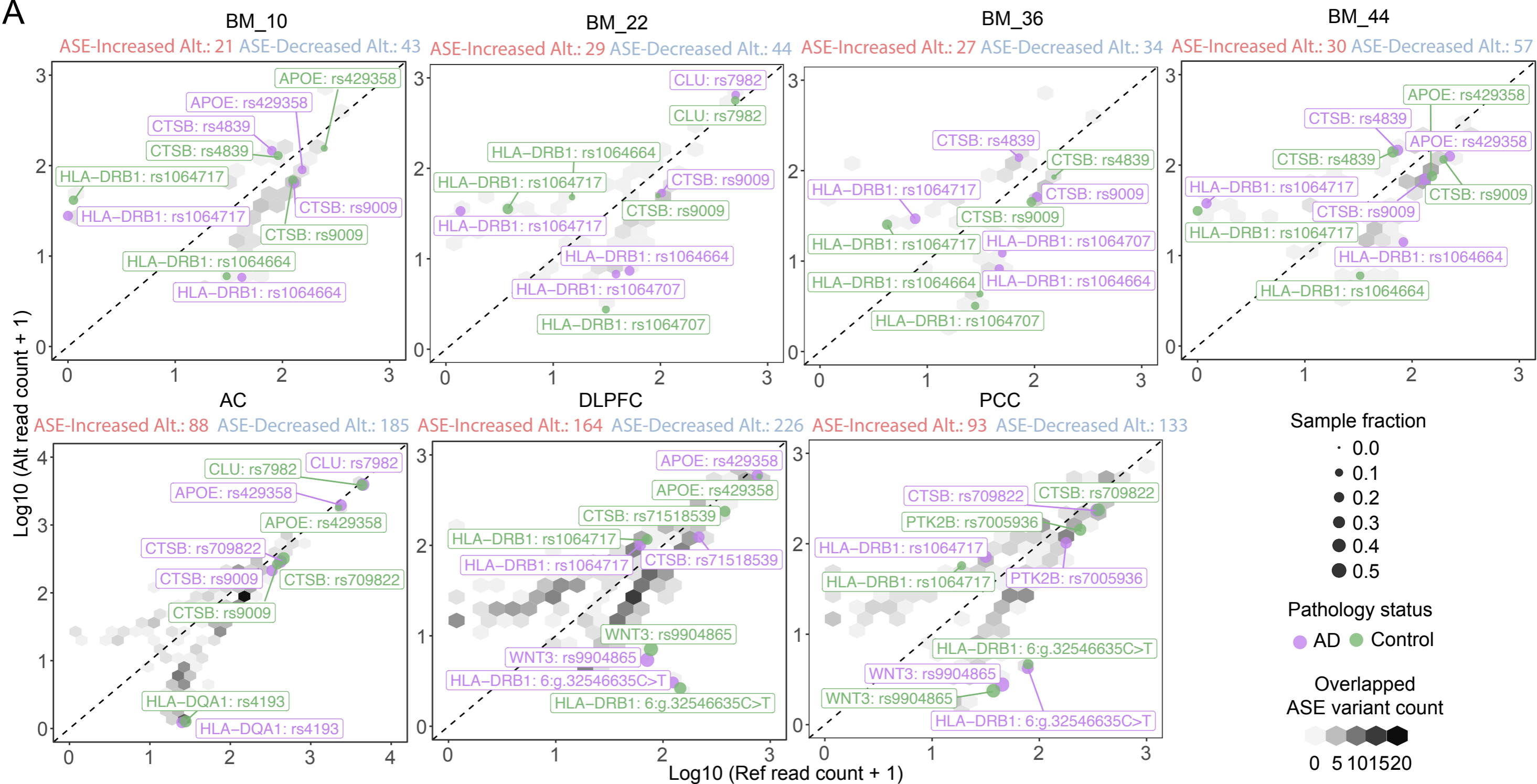

B

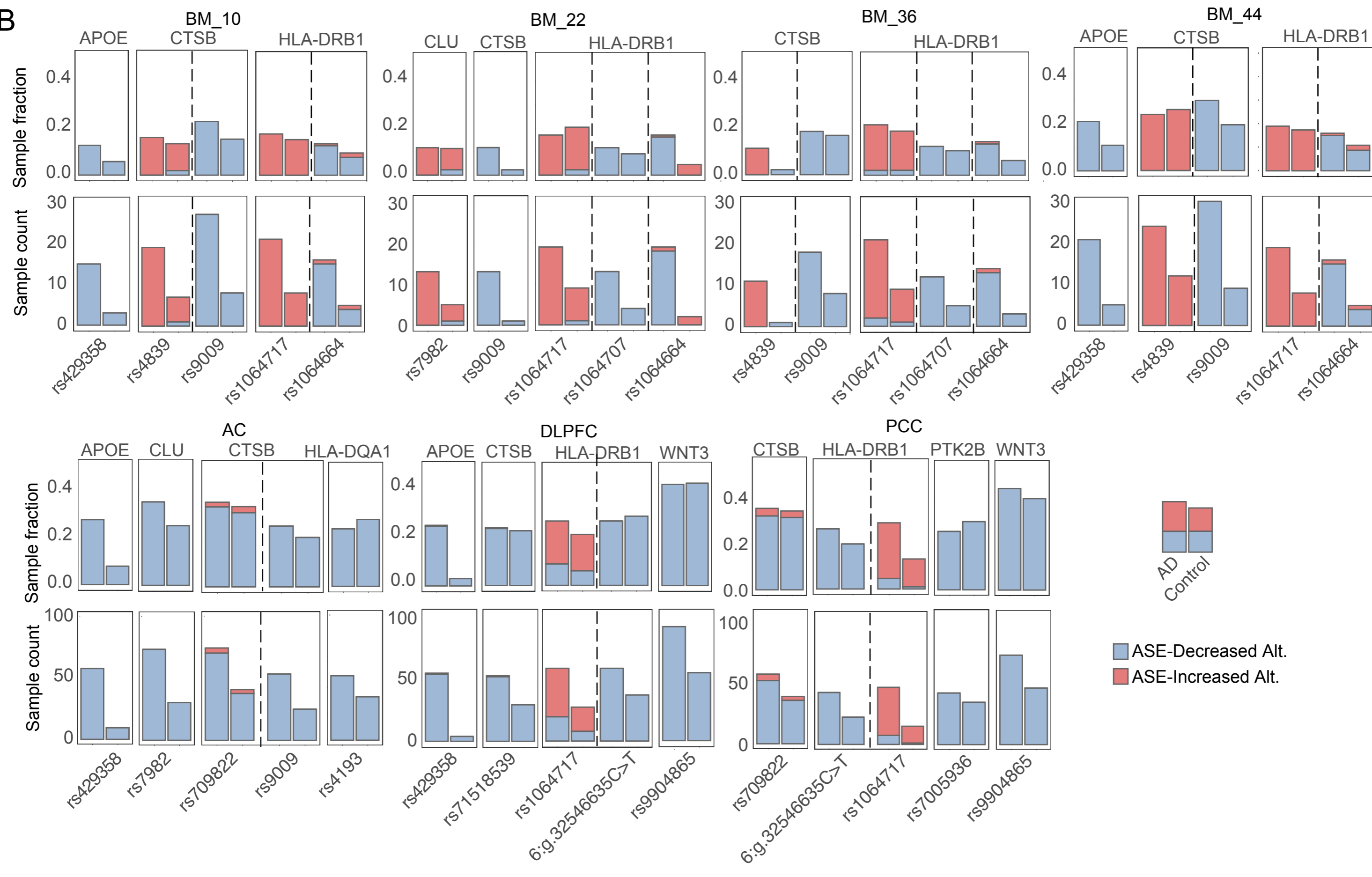

Supplement: Supplementary file 4 — Supporting Information [file ALZ-22-e71558-s006.pdf]
