# Supplementary material for: Allele specific expression in Alzheimer's disease
Source: Alzheimers Dement. 2026 Jun 11;22(6):e71558. doi: 10.1002/alz.71558 (PMC13254825; doi:10.1002/alz.71558)
Supplement: Supplementary file 5 — Supporting Information [file ALZ-22-e71558-s002.pdf]

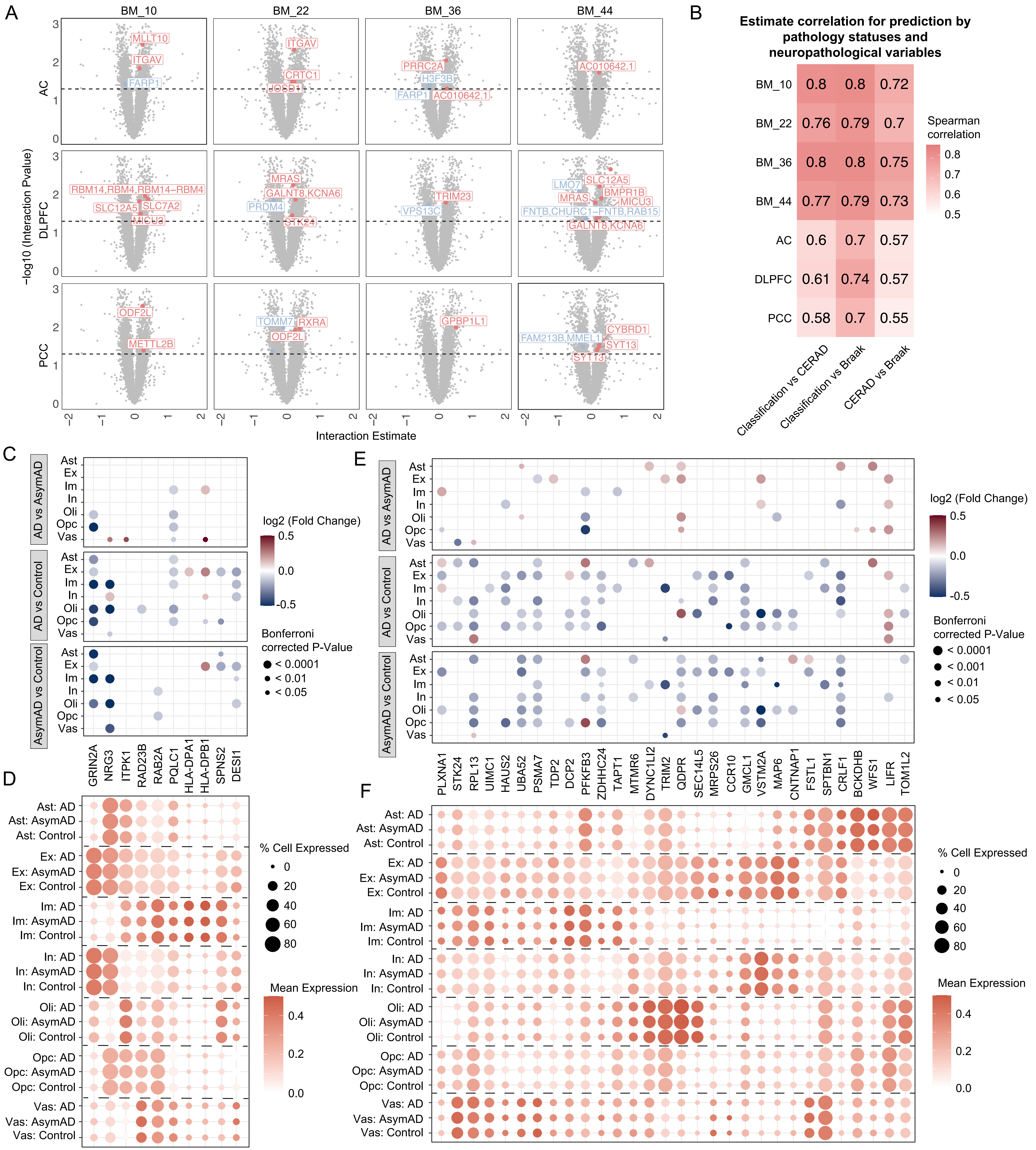
A
BM_10
BM_22
BM_36
BM_44
AC
DLPFC
PCC
−log10 (Interaction Pvalue)
Interaction Estimate
MLLT10
ITGAV
FARP1
CRTC1
JOSD1
PRRC2A
H3F3B
AC010642.1
RBM14,RBM4,RBM14−RBM4
SLC12A5
SLC7A2
MICU3
MRAS
GALNT8,KCNA6
PRDM4
STK24
TRIM23
VPS13C
LMO7
BMPR1B
FNTB,CHURC1−FNTB,RAB15
ODF2L
METTL2B
TOMM7
RXRA
GPBP1L1
CYBRD1
FAM213B,MMEL1
SYT13
B
Estimate correlation for prediction by pathology statuses and neuropathological variables
BM_10 0.8 0.8 0.72
BM_22 0.76 0.79 0.7
BM_36 0.8 0.8 0.75
BM_44 0.77 0.79 0.73
AC 0.6 0.7 0.57
DLPFC 0.61 0.74 0.57
PCC 0.58 0.7 0.55
Classification vs CERAD
Classification vs Braak
CERAD vs Braak
Spearman correlation
C
AD vs AsymAD
AD vs Control
AsymAD vs Control
Ast
Ex
Im
In
Oli
Opc
Vas
GRIN2A
NRG3
ITPK1
RAD23B
RAB2A
PQLC1
HLA-DPA1
HLA-DPB1
SPNS2
DESI1
log2 (Fold Change)
Bonferroni corrected P-Value
< 0.0001
< 0.01
< 0.05
D
Ast: AD
Ast: AsymAD
Ast: Control
Ex: AD
Ex: AsymAD
Ex: Control
Im: AD
Im: AsymAD
Im: Control
In: AD
In: AsymAD
In: Control
Oli: AD
Oli: AsymAD
Oli: Control
Opc: AD
Opc: AsymAD
Opc: Control
Vas: AD
Vas: AsymAD
Vas: Control
% Cell Expressed
Mean Expression
E
PLXNA1
STK24
RPL13
UIMC1
HAUS2
UBA52
PSMA7
TDP2
DCP2
PFKFB3
ZDHHC24
TAPT1
MTMR6
DYNC1LI2
TRIM2
QDPR
SEC14L5
MRPS26
CCR10
GMCL1
VSTM2A
MAP6
CNTNAP1
FSTL1
SPTBN1
CRLF1
BCKDHB
WFS1
LIFR
TOM1L2
< 0.001
F
